# Meat consumption and *K-ras* mutations in sporadic colon and rectal cancer in The Netherlands Cohort Study

**DOI:** 10.1038/sj.bjc.6602491

**Published:** 2005-04-05

**Authors:** M Brink, M P Weijenberg, A F P M de Goeij, G M J M Roemen, M H F M Lentjes, A P de Bruïne, R A Goldbohm, P A van den Brandt

**Affiliations:** 1 Nutrition and Toxicology Research Institute Maastricht (NUTRIM), Department of Epidemiology, Maastricht University, PO Box 616, 6200 MD, Maastricht, The Netherlands; 2 Research Institute Growth and Development (GROW), Department of Pathology, Maastricht University, PO Box 616, 6200 MD, Maastricht, The Netherlands; 3 NUTRIM, Department of Pathology, Maastricht University, The Netherlands; 4 TNO Nutrition and Food Research, Zeist, The Netherlands

**Keywords:** meat, colon, rectum, wild-type *K-ras*

## Abstract

Case–cohort analyses were performed on meat and fish consumption in relation to *K-ras* mutations in 448 colon and 160 rectal cancers that occurred during 7.3 years of follow-up, excluding the first 2.3 years, and 2948 subcohort members of The Netherlands Cohort Study on diet and cancer. Adjusted incidence rate ratios and 95% confidence intervals were computed for colon and rectal cancer and for *K-ras* mutation status subgroups. Total fresh meat, most types of fresh meat and fish were not associated with colon or rectal cancer, neither overall nor with *K-ras* mutation status. However, several weak associations were observed for tumours with a wild-type *K-ras*, including beef and colon tumours, and an inverse association for pork with colon and rectal tumours; for meat products, an increased association was observed with wild-type *K-ras* tumours in the colon and possibly with G>A transitions in rectal tumours.

Epidemiological evidence on different types of meat, meat products and fish in relation to colorectal cancer (CRC) risk is not consistent (Giovannucci and Willett, 1994; Potter, 1996; Norat *et al*, 2002) perhaps, in part, due to the heterogeneity of the colon and rectal cancer end point. Associations may become more apparent when the molecular events involved in colorectal carcinogenesis are taken into account.

Most colorectal tumours develop from small adenomatous polyps through a well-defined sequence of morphological changes (Hill *et al*, 1978), associated with the acquisition of somatic mutations (Vogelstein *et al*, 1988; Fearon and Vogelstein, 1990). A genetic alteration that occurs in adenomas (10%) as well as in carcinomas (40%) of the colorectum is the oncogenic activation of the *K-ras* gene by mutations. Activating mutations are mainly found in codons 12 and 13 (Bos *et al*, 1987; Vogelstein *et al*, 1988; Breivik *et al*, 1994; Brink *et al*, 2003); those most frequently observed are the G>A transitions, G>T and G>C transversions (Urosevic *et al*, 1993; Martinez-Garza *et al*, 1999; Brink *et al*, 2003).

The link between several types of fresh meat, meat products and fish, and the pattern of *K-ras* mutations in CRC is not clear. To date, only a few case–control and case–case studies have been conducted on the association between dietary factors and *K-ras* mutation status (Bautista *et al*, 1997; Martinez *et al*, 1999; Voskuil, 1999; Kampman *et al*, 2000; O'Brien *et al*, 2000; Slattery *et al*, 2000). Four studies reported on different types of meat consumption (Martinez *et al*, 1999; Kampman *et al*, 2000; O'Brien *et al*, 2000; Slattery *et al*, 2000).

It has been suggested that N-nitroso compounds, present in processed meat or endogenously formed (Bingham *et al*, 1996), may induce G>A transitions at the second base of codon 12 or 13 of the *K-ras* gene in the human colon (Hughes *et al*, 2001). High-temperature cooking of meat proteins generates heterocyclic aromatic amines (HAA), whereas the process of grilling and smoking of meat generates polycyclic aromatic hydrocarbons (PAH) as a result of fat dropping on open flames. HAA-DNA-adducts can cause transitions and transversions, whereas PAHs could induce G>T transversions in the *K-ras* gene. Results from relevant animal experiments, however, are not consistent (Vineis and McMichael, 1996).

Since high meat and fish consumption could contribute to acquired genetic alterations in the *K-ras* oncogene in colon and rectal tumours, these dietary items have been studied in relation to the risk of specific point mutations in the *K-ras* oncogene in colorectal cancer patients studied within The Netherlands Cohort Study on diet and cancer (NLCS).

## MATERIALS AND METHODS

### Study population

The study subjects are incident colon and rectal cancer cases and subcohort members from the NLCS, which has been described in detail elsewhere (Van den Brandt *et al*, 1990a). Briefly, the cohort study was initiated in 1986 and includes 58 279 men and 62 573 women, aged 55–69 years at baseline, who originated from 204 Dutch municipalities with computerised population registries. A self-administered questionnaire on diet and other risk factors for cancer was completed at baseline. The entire cohort is being monitored for cancer occurrence by annual record linkage to The Netherlands Cancer Registry (NCR, nine cancer registries in The Netherlands) and to PALGA, a nationwide network and registry of histo- and cytopathology (www.palga.nl) (Van den Brandt *et al*, 1990b). In the municipalities included in the NLCS, the NCR and PALGA, together, have nearly 100% coverage since the start of the study (Schouten *et al*, 1993, 1994; Van der Sanden *et al*, 1995). PALGA also provides necessary information on the identification of the pathology laboratory location of the storage of paraffin-embedded blocks of the eligible CRC patients. Accumulation of person-time in the cohort has been estimated through biennial vital status follow-up of a subcohort of 3500 men and women who were randomly selected after baseline exposure measurement. Cases with prevalent cancer other than nonmelanoma skin cancer were excluded from the subcohort, which left 3346 men and women for analyses.

The first 2.3 years of follow-up were excluded due to incomplete coverage of PALGA alone in some of the municipalities included in the NLCS. Within this period, 83 subcohort members were either deceased or diagnosed with cancer other than nonmelanoma skin cancer, leaving 3263 men and women for analysis. From 1989 till 1994, 929 incident cases with histologically confirmed CRC were observed of whom 819 could also be linked to a PALGA report of the lesion. The PALGA database was used to identify and locate tumour tissue in Dutch pathology laboratories. Colorectal cancer was classified according to site as follows: colon, that is, cecum through sigmoid colon (ICD-O-1 codes: 153.0, 153.1, 153.2, 153.3, 153.4, 153.5, 153.6, 153.7, 153.8, 153.9), rectosigmoid (ICD-O-1 code 154.0) and rectum (ICD-O-1 code 154.1). Information about age at baseline, sex and family history of CRC (at baseline) was retrieved from the NLCS database.

### Tissue samples

This study is based on data of gene mutation analysis from CRC patients, described in detail elsewhere (Brink *et al*, 2003). Briefly, tumour material of all CRC patients was collected after approval by the Medical Ethics Committees of Maastricht University, the NCR and PALGA. Subsequently, all pathology laboratories in The Netherlands agreed to make relevant tissue samples available upon request from PALGA. Tissue samples of the 819 cases were distributed among 54 pathology laboratories throughout The Netherlands. Tumour tissue collection started in August 1999 and was completed in December 2001. The loss to follow-up of tissue samples of cases amounted to 5%. Tissue samples from nine patients registered in one pathology laboratory could not be retrieved due to administrative inconsistencies, leaving 810 tissue samples for collection. For 34 cases, paraffin-embedded material was not available in the archives of pathology laboratories, leaving 776 cases for the determination of the *K-ras* mutation status. For 39 cases (5%), the *K-ras* mutation status could not be determined, that is, for 20 cases only normal colonic mucosa was available, 10 cases were revised with an benign adenoma (AdB) instead of an adenocarcinoma, for six cases the yield of DNA was not sufficient to determine *K-ras* mutation status and for three cases the available tissue did not include malignant CRC tissue. Finally, tumour material from 737 incident colorectal adenocarcinoma cases was available of whom 476 were colon cancer cases, 85 were rectosigmoid cancer cases and 176 were rectal cancer cases. Statistical analyses were performed separately for colon and rectal cancer as differences in the aetiology of colon and rectal cancer have been reported (Potter, 1996). Since the rectosigmoid can be considered as a clinically applied term rather than an anatomically defined transitional zone between the colon and rectum, patients with a rectosigmoid tumour were excluded from data-analyses. Moreover, the number of patients with a rectosigmoid tumour was too small for adequate stratified analyses (Brink *et al*, 2003).

### Detection of *K-ras* mutations

Mutation analysis of the exon 1 fragment of the *K-ras* oncogene, spanning codons 8–29, was performed on archival colorectal adenocarcinoma specimens of all 737 CRC patients using macrodissection, nested polymerase chain reaction (PCR) and direct sequencing of purified fragments, which has been described in detail elsewhere (Brink *et al*, 2003). The method of mutation detection was validated by the confirmation of reported *K-ras* status in CRC cell lines and a good correlation between fresh-frozen and routinely fixed, paraffin-embedded tissue. The detection limit was 5% mutated DNA. Duplicate analyses revealed a good reproducibility (88%) (Brink *et al*, 2003). Two observers (GR and ML) independently performed evaluation of mutation analysis and data-entry.

### The food frequency questionnaire

The dietary section of the questionnaire covered a 150-item semiquantitative food frequency, which concentrated on habitual consumption of food and beverages during the year preceding the start of the study. Daily mean nutrient intakes were calculated using the computerised Dutch food composition table (Dutch food composition table (NEVO table, 1986)), by multiplying frequencies and portion sizes of all food items with their tabulated nutrient contents. The questionnaire was validated against a 9-day diet record (Goldbohm *et al*, 1994a). The Spearman correlation coefficients for total fresh meat, meat products and fish were 0.46, 0.54 and 0.53, respectively (Goldbohm *et al*, 1994a). Questionnaire data were key-entered twice and processed for all incident cases in the cohort and for all subcohort members in a manner blinded with respect to case/subcohort status. This was carried out in order to minimise observer bias in coding and interpretation of the data.

For 257 subjects (28 incident colon adenocarcinoma cases, 16 incident rectal adenocarcinoma cases and 215 subcohort members; two subcohort members were also colon or rectal cancer cases), dietary data were incomplete or inconsistent, and they were excluded from the analyses. These subjects either (1) left 60 or more (out of 150) questionnaire items blank and ate fewer than 35 items at least once per month and/or (2) left one or more item blocks (groups of items, i.e. beverages) blank. Additional details are given elsewhere (Goldbohm *et al*, 1994a). Hence, 448 colon and 160 rectal cases and 3048 subcohort members were available for data-analyses.

The food frequency questionnaire contained 14 items on the consumption of meat with the main (hot) meal (mainly fresh meat, including chicken), five items on the consumption of meat products, which are used as sandwich filling and three items on fish consumption. For the serving size of total fresh meat, a question was included on the quantity of meat usually purchased (per person, per meal). Fresh meat is defined as meat that has not undergone some form of preservation, that is, smoking, fermentation and/or treatment with nitrate and/or nitrite salt (‘curing’) and which includes beef, pork, minced meat, chicken, liver and other meat (i.e. sausages). Coding of fresh meat items was based on raw weight to take into account the amount of fat originally present in the meat but ultimately ending up into the gravy, which is usually consumed as well. Meat products are defined as meat items that have undergone some form of preservation (mostly cured, sometimes also smoked or fermented). For chicken and fish, standard serving sizes were used. Meat items included in the questionnaire were converted into mean daily consumption in grams.

Quartiles of the consumption of total fresh meat, beef, pork, liver, minced meat, other meat and meat products were computed for men and women separately, based on the distribution of subcohort members as described in detail later. For chicken and fish, groups were classified into a nonuser and three user categories (5.3–13.2, 13.2–22.8 and ⩾22.8 g day^−1^ for chicken and 0–10, 10–20 and ⩾20 g day^−1^ for fish) and this classification was used for both men and women.

Daily intake of dietary fibre (g day^−1^), alcohol (g day^−1^), fruit (g day^−1^), vegetables (g day^−1^) and total energy (kcal day^−1^) and age at baseline (years), sex (men/women), Quetelet Index (QI; kg m^−2^), physical activity (<30 min day^−1^, 30–60 min day^−1^, 60–90 min day^−1^, >90 min day^−1^), family history of CRC (yes/no) and smoking status (never/ex/current) were regarded as potential confounders.

### Statistical analysis

The overall frequency of *K-ras* mutations as well as the type of mutation were computed for all colon and rectal cancer cases as described elsewhere (Brink *et al*, 2003). Mean values of the continuous variables age at baseline (years), consumption of total fresh meat, beef, pork, minced meat, chicken, liver, other meat, meat products, fish, dietary fibre, alcohol, fruit, vegetables, total energy and QI were evaluated for subcohort members and colon and rectal cancer cases with wild-type and mutated *K-ras* gene. Distributions of the categorical variables sex, family history of CRC, smoking status and physical activity were evaluated for subcohort members and colon and rectal cancer patients with wild-type and mutated *K-ras* gene and tested for differences between patient groups with the *χ*^2^-test. Differences in mean values of the continuous variables between patients with wild-type and mutated *K-ras* gene were tested with the Student *t*-test or the Mann–Whitney*U*-test if the variables were not normally distributed. The statistical software package SPSS (version 9) was used for these analyses.

Incidence rate ratios (RRs) and corresponding 95% confidence intervals (CI) were estimated for colon and rectal cancer cases with wild-type or mutated *K-ras* gene tumours. RRs were computed using Cox regression models with the STATA statistical software package (intercooled STATA, version 7) according to consumption of quartiles or categories and one standard deviation (s.d.) of increase in fresh meat, meat products and fish, all based on the distribution in the subcohort. The lowest quartile or category of consumption was regarded as the reference category. The person-years at risk, estimated from the subcohort, were used in the denominator of the incidence rates (Van den Brandt *et al*, 1990a). Standard errors were estimated using the robust Huber–White sandwich estimator to account for additional variance introduced by sampling from the cohort. This method is equivalent to the variance–covariance estimator as presented by Barlow *et al* (1999). The proportional hazards assumption was tested using the scaled Schoenfeld residuals (Schoenfeld, 1982). Those variables that were found to contribute substantially (*P*<0.10) to the multivariate model for colon and/or rectal cancer (age, sex, QI, smoking, energy intake and family history of CRC) were included as covariates in all multivariate analyses. Interactions between total fresh meat, meat products and fish consumption on one hand and sex on the other hand were tested for colon and rectal cancer separately and not found to be statistically significant. Therefore, results for men and women are presented together. Finally, age at baseline, sex, family history of CRC, smoking status, QI and the intake of energy were confounders for either one or both of the models, that is, with colon or rectal cancer cases, and were therefore included as covariates for all models to be tested. Since 100 subcohort members had missing values for QI, results in the tables (except for Table 1) concern 2948 subcohort members. For each analysis, linear trends were evaluated with the Wald test of the regression coefficient estimate by fitting ordinal exposure variables (quartiles/categories of consumption) as continuous terms.

## RESULTS

The overall frequency and spectrum of mutations in the *K-ras* gene have been presented in detail elsewhere (Brink *et al*, 2003). In brief, a total of 227 mutations were found in 218 (36%) out of 608 colon and rectal cancer patients. The most frequently observed mutations are the G>A transitions (54%), G>T transversions (33%) and G>C transversions (7%). The observed frequencies of the mutations in this series of patients are similar to the frequencies of the 737 CRC cases, including the rectosigmoid cancer cases, for whom *K-ras* mutation status was determined (Brink *et al*, 2003).

Table 1 shows the baseline characteristics of the study population. Colon and rectal cancer cases were more often men, were older, more frequently reported a family history of CRC, had a higher daily alcohol intake and were less frequently never smokers as compared to the subcohort. Colon cancer cases with a *K-ras* mutation in their tumour had a lower daily consumption of meat products, a higher intake of dietary fibres and were significantly older than colon cancer cases with a wild-type *K-ras* tumour (*P*-values 0.02, 0.02 and 0.006, respectively). There were no statistically significant differences between colon cancer cases with and without a *K-ras* mutation in their tumour in dietary consumption of total fresh meat, beef, pork, minced meat, liver, chicken, other meat, fish and other factors presented in Table 1. Rectal cancer cases with a *K-ras* mutation in their tumour had a lower daily consumption of minced meat, a higher daily consumption of pork and were less frequently men than rectal cancer cases with a wild-type *K-ras* gene in their tumour (*P*-values 0.03, 0.06 and 0.03, respectively). No statistically significant differences were observed between rectal cancer cases with and without a *K-ras* mutation in their tumours for other factors presented in Table 1.

Associations between the consumption of total fresh meat, different types of fresh meat, that is, beef, pork, minced meat, liver, chicken and other meat, meat products and fish, and the risk of colon or rectal cancer are presented in Table 2. Relative ratios and 95% CI for colon and rectal cancer were presented after adjustment for age and sex and after adjustment for age, sex, smoking QI, energy intake and family history of CRC. The age-and-sex adjusted RR and the multivariate RR were similar. Frequent consumption of total fresh meat, minced meat, liver, chicken, other meat, meat products and fish was not significantly associated with the risk of colon or rectal cancer (Table 2). A high intake of beef was borderline positively associated with the risk of colon cancer (RR for highest *vs* lowest quartile of consumption 1.28, 95% CI 0.96–1.72; *P*_trend_ 0.06). There was no association between beef and rectal cancer risk. A high consumption of pork was, however, borderline inversely associated with the risk of colon cancer (RR for highest *vs* lowest quartile of consumption 0.77, 95% CI 0.57–1.04; *P*_trend_ 0.07) and rectal cancer (RR for highest *vs* lowest quartile of consumption 0.70, 95% CI 0.43–1.13; *P*_trend_ 0.09).

Associations of total fresh meat, beef, pork, minced meat, liver, chicken, other meat, and meat products and fish with wild-type or mutated *K-ras* gene in colon or rectal tumours are presented in Table 3. Results will first be presented for tumours with a wild-type *K-ras* gene. No clear associations were observed for the highest *vs* the lowest consumption of total fresh meat, minced meat, liver, chicken, other meat and fish and colon and rectal tumours with a wild-type *K-ras* gene. A nonsignificant, increased association was observed between beef and wild-type *K-ras* colon tumours (RR for highest *vs* lowest quartile of consumption 1.36, 95% CI 0.96–1.93; *P*_trend_ 0.08). No association was observed for beef and rectal tumours with a wild-type *K-ras* gene. On the other hand, a high consumption of pork was inversely associated with wild-type *K-ras* colon and rectal tumours (RR for highest *vs* lowest quartile of consumption 0.72, 95% CI 0.51–1.02; *P*_trend_ 0.05 and 0.50, 95% CI 0.26–0.93; *P*_trend_ 0.01, respectively). A high consumption of meat products showed a significant increased association with colon tumours with a wild-type *K-ras* gene (RR for highest *vs* lowest quartile of intake 1.42, 95% CI 1.00–2.03; *P*_trend_ 0.03). No clear association was found for high consumption of meat products and rectal tumours with a wild-type *K-ras* gene. Regarding colon and rectal tumours that harbour a *K-ras* mutation, no clear associations were observed between highest *vs* lowest quartile of consumption of different types of fresh meat, meat products and fish.

Subgroup analyses were performed to evaluate the associations between total fresh meat, different types of fresh meat, meat products and fish, and specific types of *K-ras* point mutations (G>A transitions and G>T or G>C transversions) in colon and rectal tumours (Table 4). Results will first be presented for tumours with a G>A transition in the *K-ras* gene. Total fresh meat consumption was not associated with G>A transitions in the colon tumours, but an inverse association with G>A transitions in the rectal tumours was observed. However, none of the specific types of fresh meat, that is, beef, pork, minced meat, liver, chicken, other meat, nor fish were associated with the risk of G>A transitions in both colon and rectal tumours. For meat products, a borderline significant trend with increased risk of rectal tumours harbouring G>A transitions was observed (RR for highest *vs* lowest quartile of intake 2.37, 95% CI 0.75–7.51; *P*_trend_ 0.07). Regarding G>T or G>C transversions in colon and rectal tumours, no clear associations were observed for total fresh meat, the different types of fresh meat, meat products and fish.

## DISCUSSION

In this large cohort study with 448 incident colon and 160 incident rectal cancer patients, no associations were observed between total fresh meat and fish, and the risk of colon or rectal cancer, either overall or after *K-ras* mutation status was taken into account. This was also observed for specific types of fresh meat. However, several weak associations were observed regarding tumours harbouring a wild-type *K-ras* gene. An increased association for high consumption of beef and an inverse association for high consumption of pork, and the risk of colon tumours were observed. In addition, consumption of pork was inversely associated with rectal tumours with a wild-type *K-ras* gene. For meat products, an increased association was observed with wild-type *K-ras* tumours in the colon and a nonsignificant positive association with G>A transitions in the *K-ras* gene in rectal tumours.

Earlier results on meat and CRC in the NLCS, based on 3.3 years of follow-up, showed no association for high consumption of total fresh meat and fish and colon cancer risk in men and women. A positive association for both men and women was observed for meat products, which consist mainly of cured meat (Goldbohm *et al*, 1994b). Rectal cancer was not included in these analyses. These findings were in line with the findings of a meta-analysis conducted by Norat *et al* (2002). In total, 29 studies were evaluated, including 22 case–control and seven cohort studies for investigating the association between processed meat and CRC. A high consumption of processed meat was found to be associated with a moderate, but significant, increase in CRC risk. In the current study, after 7.3 years of follow-up and with the exclusion of the first 2.3 years, these findings were similar to the earlier results on meat and CRC in the NLCS, that is, neither an association was observed for total fresh meat, different types of fresh meat, nor for fish. However, in the current study, no significant association was observed between meat products and colon or rectal cancer risk overall.

With regard to *K-ras* mutations, two case–control studies with colon cancer patients, one with 2418 patients and 2410 controls (Slattery *et al*, 2000), the other with 185 patients (Voskuil, 1999; Kampman *et al*, 2000), and one cross-sectional case–case study with rectal cancer patients (O'Brien *et al*, 2000) have previously been conducted and have reported on the association between meat consumption and *K-ras* mutations. In none of these studies an association between meat consumption and colon or rectal cancer was observed after the *K-ras* gene mutation status was taken into account. The current cohort study on total fresh meat, different types of fresh meat, meat products and fish and specific *K-ras* mutations is, to our knowledge, the only prospective study performed to date. The prospective design of this study and the high completeness of follow-up of cancer incidence and of the subcohort make information and selection bias unlikely. In addition, as a result of the exclusion of the first 2.3 years of follow-up, the chance of information bias due to potential preclinical colorectal cancer is minimal.

Epidemiological studies have indicated that consumption of broiled, fried, barbequed or smoked meats may increase the risk of CRC (Gerhardsson de Verdier *et al*, 1991; Augustsson *et al*, 1999; Kampman *et al*, 1999), although these findings were not consistent. Augustsson *et al* (1999) observed an inverse association between high intake of HAA and the risk of colon and rectal cancer. However, Gerhardsson de Verdier *et al* (1991) observed an increased association for total meat intake as well as frequent consumption of brown gravy and a preference for a heavily browned meat surface and the risk of CRC. The relative risks (RR) were higher for rectal than for colon cancer. High-temperature cooking of meat proteins generates HAA, whereas the process of grilling and smoking of meat forms PAH as a result of fat dropping on open flames. Animal and *in vitro* studies have shown that HAA-DNA-adducts can cause transitions and transversions, as observed in the *K-ras* oncogene. However, results from animal experiments are not entirely consistent (Vineis and McMichael, 1996). In humans, a higher prevalence of G>T transversions in the *K-ras* gene was observed in 37 colorectal tumours from Yugoslavia, which the authors explain as possibly being due to the extensive use of barbequed and smoked meat throughout the year in Yugoslavia (Urosevic *et al*, 1993). Unfortunately, information on meat preparation, including cooking methods, was not collected at baseline in the current study. Since fresh meat generally needs further preparation, this type of meat may be an important source of these carcinogens. Hence, it is plausible that associations between fresh meat and colon or rectal cancer with specific point mutations exist. However, in this study, no associations were found between any of the fresh meat variables and risk of colon or rectal cancer with specific point mutations. This could be due to the expected low content of carcinogens in prepared fresh meat consumed in this cohort, and also to the lack of correlation between meat preparation and the amount of fresh meat consumed. Surprisingly, associations were observed between beef, pork and meat products and cancer with a wild-type *K-ras* gene. Possibly beef, pork and meat products exert their action through another pathway than the *K-ras* signalling route. Although these associations were weak and the inverse association for high consumption of pork cannot readily be explained, they are intriguing and require replication and further study.

It is hypothesised that fat content of meat could influence CRC risk by increasing the excretion of bile acids (Norat *et al*, 2002). The products of the bile acid excretion may act as tumour promoters by a nonspecific effect that increases cell proliferation in the mucosa layer (Norat *et al*, 2002). However, total fat intake (Brink *et al*, 2004) as well as total fresh meat consumption was not associated with overall colon and rectal cancer risk nor with *K-ras* mutation status. In the previous study (Brink *et al*, 2004), only *ω*-6 polyunsaturated fat (PUFA) was observed to be associated with specific *K-ras* mutations; however, this type of fat is not predominantly present in meat.

Nitrosamines and their precursors are compounds observed in red and processed meat (Mirvish *et al*, 2002) or can be endogenously formed (Bingham *et al*, 2002). Alkylating DNA agents like nitrosamines could generate *O*^6^-methylguanines and these have been detected in human colonic tissue (Hall *et al*, 1991). N-nitroso compounds could also induce G>A transitions in codons 12 or 13 of the *K-ras* gene of rat colon carcinomas (Zarbl *et al*, 1985; Topal, 1988; Jacoby *et al*, 1992) or in human colonic tissue (Hughes *et al*, 2001; Bingham *et al*, 2002). In the current study, no clear association was observed for daily consumption of meat products and overall colon and rectal cancer risk. When the absence or presence of *K-ras* mutations was taken into account, a high intake of meat products was found to be positively associated with colon tumours with a wild-type *K-ras* gene, as discussed above. In contrast, subgroup analysis of specific point mutations in the *K-ras* gene showed that high consumption of meat products is positively associated with rectal tumours harbouring a G>A transition. Although the association was not statistically significant, this observation is in line with the biological evidence (Zarbl *et al*, 1985; Topal, 1988; Jacoby *et al*, 1992; Hughes *et al*, 2001; Bingham *et al*, 2002). Why the association in our study is confined to the rectum and not to the colon remains unclear. A plausible explanation for differences in tumour site could be due to the duration of contact with, and the concentration of, the potential dietary carcinogens like nitrosamines. Possibly, the lower transit time of stool in the rectum as compared to the colon leads to an increased exposure time for the rectum. On the other hand, this could be a chance finding, especially concerning the observation that no association was observed between meat products and rectal cancer overall nor with the *K-ras* mutation status Therefore, more aetiological insight in the underlying mechanisms is required to clarify this issue.

We acknowledge that multiple comparisons were performed so that some of the observed associations are chance findings. Therefore, caution is warranted in interpreting the results.

In conclusion, our results indicate that total fresh meat and fish are not associated with colon or rectal cancer risk or with the *K-ras* mutation status of these cancer types. However, consumption of beef, pork and meat products appear to be associated with colon or rectal tumours with a wild-type *K-ras* gene, suggesting that they may exert their actions in colon or rectal cancer through a pathway independent of a mutation in the *K-ras* gene.

## Figures and Tables

**Table 1:** Meat variables and other characteristics (mean±s.d.) of the study population at baseline

		**Colon cancer**			**Rectal cancer**		
	**Subcohort**	**Wild-type *K-ras*^−^**	**Mutated *K-ras*^+^^b^**	***P*-value^a^**	**Wild-type *K-ras*^−^**	**Mutated *K-ras*^+^^c^**	***P*-value^a^**
*N*	3048	297	151		93	67	
Sex (% men)	48.4	51.5	58.9	*0.14*	72.0	55.2	*0.03*
Age (years)	61.4±4.2	62.7±4.0	63.8±4.1	*0.006*	62.6±4.1	62.2±4.0	*0.50*

*Meat variables*							
Total fresh meat (g day^−1^)	99.7±42.2	98.4±37.0	99.3±39.1	*0.82*	100.6±40.3	95.7±36.6	*0.43*
Beef (g day^−1^)	25.7±22.5	27.9±21.8	27.6±23.6	*0.90*	27.6±24.6	22.5±23.3	*0.19*
Pork (g day^−1^)	38.4±30.3	36.3±30.1	37.6±29.6	*0.67*	32.2±27.0	40.8±29.3	*0.06*
Minced meat (g day^−1^)	18.3±17.3	17.2±13.4	18.0±16.0	*0.57*	23.2±25.2	16.7±12.4	*0.03*
Liver (g day^−1^)	2.0±4.4	1.9±4.2	1.8±4.1	*0.29*	2.1±4.1	1.9±4.4	*0.90*
Chicken (g day^−1^)	13.7±15.6	13.2±15.5	12.4±15.2	*0.55*	14.5±15.6	12.3±12.8	*0.62*
Other meat (g day^−1^)	2.5±6.4	2.9±8.0	2.7±5.9	*0.95*	2.2±4.1	2.7±4.8	*0.84*
Meat products (g day^−1^)	13.2±15.0	15.0±16.6	11.7±13.2	*0.02*	13.5±14.7	15.0±12.9	*0.51*
Fish and shellfish (g day^−1^)	12.7±15.0	11.4±13.5	13.2±15.7	*0.24*	12.4±13.1	11.0±10.3	*0.71*

*Other dietary factors*							
Fibre (g day^−1^)	27.0±8.2	26.7±7.6	27.7±8.8	*0.02*	27.8±8.0	27.6±7.8	*0.87*
Alcohol (g day^−1^)	10.1±14.1	11.0±15.4	10.8±14.2	*0.59*	13.8±17.6	11.2±12.2	*0.30*
Fruit (g day^−1^)	176.3±117.7	172.7±123.7	176.9±122.2	*0.73*	184.3±145.8	180.7±129.9	*0.87*
Vegetable (g day^−1^)	193.6±82.3	183.3±78.2	198.0±87.0	*0.07*	192.9±72.4	188.0±110.6	*0.74*
Energy (kcal day^−1^)	1919.4±518.1	1916.5±494.4	1902.7±472.9	*0.78*	2027.3±517.4	1997.0±449.1	*0.70*

*Other characteristics*							
Quetlet Index (kg m^−2^)	25.1±3.1	25.5±3.2	25.8±3.3	*0.42*	24.9±2.8	25.5±2.9	*0.20*
Family history of CRC (% yes)	5.6	13.5	9.3	*0.20*	9.7	11.9	*0.66*
Smoker (%)							
Never	37.0	36.7	37.1		25.8	34.3	
Ex smoker	35.2	43.1	46.4		41.9	43.3	
Current smoker	27.8	20.2	16.6	*0.62*	32.3	22.4	*0.31*
*Physical activity* (%)^d^							
<30 min day^−1^	20.9	19.7	20.3		18.5	22.7	
30–60 min day^−1^	32.7	33.7	32.4		27.2	27.3	
60–90 min day^−1^	30.9	29.6	29.1		32.6	34.8	
>90 min day^−1^	15.5	17.0	18.2	*0.98*	21.7	15.2	*0.73*

a Comparing cases with at least one *K-ras* mutation to cases without a *K-ras* mutation.

b Six out of 151 colon cancer cases had more than one *K-ras* mutation.

c Three out of 67 rectal cancer cases had more than one *K-ras* mutation.

d For 41 subcohort members and nine colorectal cancer cases, information on physical activity was not available.

**Table 2:** Incidence rate ratios (RRs) and 95% confidence intervals (CI) for colon (*N*=448) and rectal (*N*=160) cancer patients overall according to the intake of total fresh meat, meat products and fish

	**Quartile/category of intake**						
**Exposure**		**1^a^**	**2**	**3**	**4**	***P* for trend**	**RR (95% CI) for one** **s.d. increase in intake^b^**
*Total fresh meat*							
Median intake (g day^−1^)	Men	61	91	110.7	150.8		
	Women	50.7	80.3	103.4	139.2		
*Cases*	*Colon*	*109*	*112*	*123*	*104*		
	*Rectum*	*40*	*48*	*40*	*32*		
*Person-years*		*3661*	*3701*	*3660*	*3715*		
RR (95% CI)^c^	Colon	1	1.03 (0.77–1.37)	1.16 (0.88–1.54)	0.99 (0.74–1.33)	*0.8*	0.97 (0.89–1.07)
	Rectum	1	1.21 (0.78–1.86)	1.03 (0.65–1.61)	0.82 (0.51–1.33)	*0.33*	0.94 (0.80–1.09)
RR (95% CI)^d^	Colon	1	1.05 (0.78–1.41)	1.12 (0.83–1.49)	0.96 (0.70–1.31)	*0.92*	0.96 (0.87–1.06)
	Rectum	1	1.11 (0.71–1.73)	0.93 (0.59–1.47)	0.72 (0.44–1.19)	*0.14*	0.89 (0.76–1.05)

*Beef*							
Median intake (g day^−1^)	Men	4.1	16.1	30	51.4		
	Women	3	14	25.5	46.9		
*Cases*	*Colon*	*100*	*98*	*108*	*142*		
	*Rectum*	*39*	*49*	*32*	*40*		
*Person-years*		*3648*	*3742*	*3507*	*3840*		
RR (95% CI)^c^	Colon	1	0.94 (0.70–1.27)	1.08 (0.80–1.44)	1.23 (0.93–1.63)	*0.09*	1.05 (0.96–1.15)
	Rectum	1	1.22 (0.79–1.88)	0.84 (0.52–1.37)	0.90 (0.57–1.43)	*0.35*	0.95 (0.79–1.13)
RR (95% CI)^d^	Colon	1	0.98 (0.72–1.33)	1.09 (0.81–1.48)	1.28 (0.96–1.72)	*0.06*	1.06 (0.97–1.17)
	Rectum	1	1.25 (0.80–1.96)	0.82 (0.49–1.36)	0.92 (0.57–1.49)	*0.38*	0.94 (0.78–1.14)

*Pork*							
Median intake (g day^−1^)	Men	7.9	28	44.3	76		
	Women	5	22.5	40.1	66.2		
*Cases*	*Colon*	*121*	*120*	*109*	*98*		
	*Rectum*	*44*	*46*	*36*	*34*		
*Person-years*		*3659*	*3658*	*3688*	*3733*		
RR (95% CI)^c^	Colon	1	1.03 (0.78–1.35)	0.94 (0.71–1.24)	0.86 (0.76–1.37)	*0.24*	0.96 (0.87–1.07)
	Rectum	1	1.07 (0.70–1.64)	0.84 (0.53–1.33)	0.80 (0.50–1.28)	*0.22*	0.90 (0.76–1.07)
RR (95% CI)^d^	Colon	1	0.98 (0.74–1.30)	0.90 (0.67–1.20)	0.77 (0.57–1.04)	*0.07*	0.93 (0.83–1.04)
	Rectum	1	1.01 (0.66–1.56)	0.81 (0.51–1.28)	0.70 (0.43–1.13)	*0.09*	0.87 (0.72–1.03)

*Minced meat*							
Median intake (g day^−1^)	Men	3.2	11.5	21	37.8		
	Women	0	9.6	18.1	32.9		
*Cases*	*Colon*	*104*	*119*	*128*	*97*		
	*Rectum*	*33*	*38*	*54*	*35*		
*Person-years*		*3648*	*3718*	*3692*	*3679*		
RR (95% CI)^c^	Colon	1	1.12 (0.84–1.49)	1.28 (0.97–1.70)	0.95 (0.70–1.28)	*0.99*	0.95 (0.86–1.04)
	Rectum	1	1.13 (0.70–1.82)	1.67 (1.07–2.61)	1.07 (0.66–1.74)	*0.38*	1.09 (0.94–1.26)
RR (95% CI)^d^	Colon	1	1.11 (0.83–1.50)	1.26 (0.94–1.69)	0.93 (0.68–1.27)	*0.88*	0.95 (0.85–1.05)
	Rectum	1	1.06 (0.66–1.73)	1.60 (1.01–2.52)	1.01 (0.62–1.67)	*0.5*	1.08 (0.92–1.27)

*Liver*							
Median intake (g day^−1^)	Men	0	4.1				
	Women	0	3.7				
*Cases*	*Colon*	*295*	*153*				
	*Rectum*	*99*	*61*				
*Person-years*		*9589*	*5148*				
RR (95% CI)^c^	Colon	1	1.02 (0.83–1.26)			*0.84*	1.00 (0.91–1.11)
	Rectum	1	1.16 (0.83–1.62)			*0.38*	1.02 (0.88–1.18)
RR (95% CI)^d^	Colon	1	1.04 (0.84–1.29)			*0.71*	1.02 (0.92–1.12)
	Rectum	1	1.11 (0.79–1.57)			*0.54*	1.02 (0.88–1.18)

*Chicken*							
Median intake (g day^−1^)	Men/women	0	5.3	13.2	22.8		
*Cases*	*Colon*	*125*	*101*	*98*	*124*		
	*Rectum*	*35*	*44*	*33*	*48*		
*Person-years*		*3457*	*3564*	*3616*	*4100*		
RR (95% CI)^c^	Colon	1	0.81 (0.61–1.07)	0.78 (0.58–1.04)	0.86 (0.66–1.13)	*0.3*	0.96 (0.86–1.07)
	Rectum	1	1.26 (0.80–2.00)	0.92 (0.56–1.50)	1.18 (0.76–1.86)	*0.77*	1.00 (0.86–1.16)
RR (95% CI)^d^	Colon	1	0.85 (0.63–1.14)	0.81 (0.60–1.08)	0.87 (0.66–1.15)	*0.34*	0.95 (0.85–1.07)
	Rectum	1	1.26 (0.79–2.03)	0.94 (0.57–1.55)	1.12 (0.70–1.79)	*0.96*	0.98 (0.83–1.14)

*Other meat*							
Median intake (g day^−1^)	Men	0	6.6				
	Women	0	7				
*Cases*	*Colon*	*313*	*135*				
	*Rectum*	*105*	*55*				
*Person-years*		*10 421*	*4316*				
RR (95% CI)^c^	Colon	1	1.13 (0.91–1.41)			*0.25*	1.05 (0.96–1.15)
	Rectum	1	1.29 (0.91–1.81)			*0.15*	0.98 (0.86–1.11)
RR (95% CI)^d^	Colon	1	1.16 (0.92–1.46)			*0.2*	1.08 (0.98–1.18)
	Rectum	1	1.34 (0.95–1.91)			*0.1*	1.00 (0.88–1.15)

*Meat products* e							
Median intake (g day^−1^)	Men	1	7.4	15.8	33.3		
	Women	0	4.3	10.5	22.4		
*Cases*	*Colon*	*113*	*94*	*118*	*123*		
	*Rectum*	*44*	*30*	*39*	*47*		
*Person-years*		*3588*	*3624*	*3794*	*3732*		
RR (95% CI)^c^	Colon	1	0.87 (0.65–1.17)	1.06 (0.80–1.40)	1.13 (0.86–1.49)	*0.22*	1.04 (0.95–1.14)
	Rectum	1	0.70 (0.43–1.12)	0.88 (0.57–1.38)	1.09 (0.71–1.66)	*0.54*	1.01 (0.89–1.16)
RR (95% CI)^d^	Colon	1	0.89 (0.65–1.21)	1.06 (0.78–1.42)	1.17 (0.86–1.59)	*0.19*	1.05 (0.94–1.16)
	Rectum	1	0.68 (0.41–1.11)	0.84 (0.53–1.35)	1.04 (0.64–1.68)	*0.7*	0.97 (0.84–1.13)

*Fish*							
Median intake (g day^−1^)	Men	0	4.6	14.8	30.5		
	Women	0	4.6	15.5	28.2		
*Cases*	*Colon*	*123*	*122*	*116*	*87*		
	*Rectum*	*48*	*33*	*48*	*31*		
*Person-years*		*4235*	*3238*	*4444*	*2820*		
RR (95% CI)^c^	Colon	1	1.31 (1.00–1.73)	0.92 (0.70–1.21)	1.05 (0.78–1.41)	*0.64*	0.94 (0.85–1.04)
	Rectum	1	0.86 (0.54–1.36)	0.93 (0.62–1.41)	0.89 (0.56–1.42)	*0.7*	0.91 (0.79–1.06)
RR (95% CI)^d^	Colon	1	1.30 (0.97–1.73)	0.82 (0.62–1.09)	1.03 (0.76–1.40)	*0.4*	0.93 (0.83–1.04)
	Rectum	1	0.88 (0.55–1.43)	0.97 (0.64–1.48)	0.94 (0.59–1.52)	*0.89*	0.93 (0.80–1.08)

a Reference category/quartile of intake.

b See for one s.d. of increase based on the intake of the subcohort (Table 1).

c Rate ratios adjusted for age and sex.

d Rate ratios adjusted for age, sex, Quetelet Index (QI), smoking, energy intake and family history of colorectal cancer (CRC).

e Rate ratios per increment of 15 g day^−1^ (s.d. in subcohort), equivalent to one standard sandwich filling.

**Table 3:** Adjusted RR^a^ for colon and rectal cancer patients with a *K-ras* mutation status^b^ according to the intake of fresh meat, meat products and fish

	**Quartile/category of intake**						
**Exposure**		**1^c^**	**2**	**3**	**4**	***P* for trend**	**RR (95% CI) for one** **s.d. increase in intake^d^**
*Total fresh meat*							
*Cases*							
*K-ras*^−^*/K-ras*^+^	*Colon*	*68*/*35*	*80*/*29*	*76*/*45*	*66*/*35*		
	*Rectum*	*25*/*15*	*21*/*24*	*25*/*14*	*18*/*12*		
RR_*K-ras*−_ (95% CI)	Colon	1	1.16 (0.82–1.64)	1.05 (0.74–1.49)	0.94 (0.65–1.36)	*0.61*	0.95 (0.84–1.07)
	Rectum	1	0.85 (0.47–1.55)	1.02 (0.58–1.80)	0.76 (0.41–1.44)	*0.56*	0.93 (0.75–1.15)
RR_*K-ras*+_ (95% CI)	Colon	1	0.83 (0.50–1.37)	1.25 (0.79–2.00)	1.01 (0.61–1.66)	*0.57*	0.99 (0.85–1.16)
	Rectum	1	1.53 (0.80–2.94)	0.82 (0.39–1.72)	0.68 (0.31–1.47)	*0.1*	0.85 (0.67–1.07)
*Beef*							
*Cases*							
*K-ras*^−^*/K-ras*^+^	*Colon*	*62/32*	*66*/*29*	*66*/*39*	*96*/*44*		
	*Rectum*	*21/16*	*25*/*23*	*18*/*12*	*25*/*14*		
RR_*K-ras*−_ (95% CI)	Colon	1	1.04 (0.72–1.49)	1.05 (0.72–1.51)	1.36 (0.96–1.93)	*0.08*	1.08 (0.97–1.20)
	Rectum	1	1.16 (0.64–2.12)	0.89 (0.46–1.71)	1.06 (0.57–1.94)	*0.93*	1.02 (0.81–1.27)
RR_*K-ras*+_ (95% CI)	Colon	1	0.87 (0.52–1.47)	1.18 (0.73–1.92)	1.14 (0.71–1.83)	*0.38*	1.04 (0.89–1.22)
	Rectum	1	1.36 (0.70–2.63)	0.74 (0.34–1.61)	0.75 (0.35–1.61)	*0.2*	0.84 (0.61–1.16)
*Pork*							
*Cases*							
*K-ras*^−^*/K-ras*^+^	*Colon*	*85/33*	*74*/*42*	*68*/*40*	*63*/*29*		
	*Rectum*	*31/12*	*25*/*19*	*18*/*18*	*15*/*16*		
RR_*K-ras*−_ (95% CI)	Colon	1	0.86 (0.61–1.20)	0.77 (0.55–1.08)	0.72 (0.51–1.02)	*0.05*	0.91 (0.80–1.05)
	Rectum	1	0.81 (0.48–1.39)	0.58 (0.32–1.04)	0.50 (0.26–0.93)	*0.01*	0.74 (0.57–0.96)
RR_*K-ras*+_ (95% CI)	Colon	1	1.30 (0.80–2.09)	1.24 (0.76–2.02)	0.90 (0.53–1.53)	*0.68*	0.97 (0.82–1.15)
	Rectum	1	1.52 (0.73–3.17)	1.38 (0.65–2.94)	1.21 (0.56–2.60)	*0.75*	1.03 (0.82–1.29)
*Minced meat*							
*Cases*							
*K-ras*^−^*/K-ras*^+^	*Colon*	*66/34*	*79*/*37*	*89*/*36*	*56*/*37*		
	*Rectum*	*19/14*	*17*/*19*	*32*/*20*	*21*/*12*		
RR_*K-ras*−_ (95% CI)	Colon	1	1.16 (0.82–1.64)	1.37 (0.97–1.92)	0.85 (0.58–1.25)	*0.68*	0.93 (0.82–1.04)
	Rectum	1	0.89 (0.46–1.74)	1.77 (0.99–3.19)	1.17 (0.62–2.19)	*0.21*	1.18 (0.99–1.40)
RR_*K-ras*+_ (95% CI)	Colon	1	1.04 (0.64–1.68)	1.06 (0.64–1.74)	1.07 (0.65–1.75)	*0.78*	0.98 (0.83–1.16)
	Rectum	1	1.28 (0.64–2.56)	1.36 (0.68–2.72)	0.81 (0.37–1.79)	*0.66*	0.87 (0.68–1.12)
Liver							
*Cases*							
*K-ras*^−^*/K-ras*^+^	*Colon*	*184/98*	*106*/*46*				
	*Rectum*	*55/41*	*34*/*24*				
RR_*K-ras*−_ (95% CI)	Colon	1	1.10 (0.86–1.42)			*0.44*	1.02 (0.91–1.14)
	Rectum	1	1.15 (0.74–1.79)			*0.54*	1.05 (0.89–1.24)
RR_*K-ras*+_ (95% CI)	Colon	1	0.92 (0.63–1.33)			*0.65*	1.01 (0.85–1.19)
	Rectum	1	1.07 (0.63–1.80)			*0.8*	0.97 (0.74–1.28)
*Chicken*							
*Cases*							
*K-ras*^−^*/K-ras*^+^	*Colon*	*73/42*	*73*/*27*	*62*/*35*	*82*/*40*		
	*Rectum*	*20/13*	*24*/*19*	*16*/*17*	*29*/*16*		
RR_*K-ras*−_ (95% CI)	Colon	1	0.96 (0.68–1.36)	0.81 (0.57–1.16)	0.91 (0.65–1.28)	*0.44*	0.96 (0.84–1.09)
	Rectum	1	1.19 (0.64–2.18)	0.76 (0.39–1.49)	1.23 (0.69–2.21)	*0.75*	1.04 (0.85–1.26)
RR_*K-ras*+_ (95% CI)	Colon	1	0.64 (0.39–1.06)	0.80 (0.51–1.28)	0.80 (0.51–1.26)	*0.52*	0.95 (0.79–1.14)
	Rectum	1	1.39 (0.68–2.85)	1.20 (0.57–2.53)	0.97 (0.46–2.03)	*0.75*	0.88 (0.68–1.15)
*Other meat*							
*Cases*							
*K-ras*^−^*/K-ras*^+^	Colon	*201/101*	*89*/*43*				
	Rectum	*57/43*	*32*/*22*				
RR_*K-ras*−_ (95% CI)	Colon	1	1.16 (0.89–1.52)			*0.28*	1.08 (0.96–1.21)
	Rectum	1	0.90 (0.63–2.23)			*0.14*	0.97 (0.81–1.17)
RR_*K-ras*+_ (95% CI)	Colon	1	1.16 (0.79–1.70)			*0.44*	1.07 (0.93–1.23)
	Rectum	1	1.24 (0.73–2.10)			*0.43*	1.04 (0.87–1.24)
*Meat products* e							
*Cases*							
*K-ras*^−^*/K-ras*^+^	*Colon*	*65/40*	*59*/*33*	*76*/*41*	*90*/*30*		
	*Rectum*	*28/14*	*20*/*9*	*18*/*20*	*23*/*22*		
RR_*K-ras*−_ (95% CI)	Colon	1	0.91 (0.63–1.33)	1.10 (0.76–1.57)	1.42 (1.00–2.03)	*0.03*	1.11 (0.99–1.25)^e^
	Rectum	1	0.71 (0.39–1.30)	0.62 (0.34–1.16)	0.84 (0.45–1.58)	*0.51*	0.91 (0.73–1.14)^e^
RR_*K-ras*+_ (95% CI)	Colon	1	0.84 (0.52–1.37)	1.00 (0.63–1.59)	0.77 (0.45–1.32)	*0.51*	0.90 (0.73–1.11)^e^
	Rectum	1	0.62 (0.26–1.46)	1.26 (0.61–2.61)	1.41 (0.67–2.99)	*0.17*	1.05 (0.86–1.27)^e^

Fish							
*Cases*							
*K-ras*^−^*/K-ras*^+^	*Colon*	*84/37*	*81*/*36*	*74*/*35*	*51*/*36*		
	*Rectum*	*25/19*	*20*/*11*	*24*/*24*	*20*/*11*		
RR_*K-ras*−_ (95% CI)	Colon	1	1.30 (0.93–1.81)	0.80 (0.57–1.12)	0.87 (0.60–1.26)	*0.13*	0.88 (0.77–1.02)
	Rectum	1	0.97 (0.53–1.79)	0.86 (0.48–1.52)	1.06 (0.58–1.94)	*0.99*	0.97 (0.81–1.17)
RR_*K-ras*+_ (95% CI)	Colon	1	1.29 (0.79–2.10)	0.87 (0.54–1.42)	1.38 (0.85–2.25)	*0.52*	1.00 (0.85–1.19)
	Rectum	1	0.76 (0.35–1.61)	1.13 (0.61–2.07)	0.78 (0.37–1.65)	*0.84*	0.86 (0.69–1.07)

a Multivariate adjusted rate ratios (RRs) for age, sex, Quetelet Index (QI), smoking, energy intake and family history of CRC and their 95% confidence intervals.

b Wild-type *K-ras* (*K-ras*^−^): no mutation in the exon 1 fragment of the gene, mutated *K-ras* (*K-ras^+^)*: at least one mutation in the exon 1 fragment of the gene.

c Reference category/quartile of intake.

d See for one s.d. of increase based on the intake of the subcohort (Table 1).

e Rate ratios per increment of 15 g day^−1^ (s.d. in subcohort), equivalent to one standard sandwich filling.

**Table 4:** Adjusted RR^a^ for colon and rectal cancer patients with specific point mutations^b^ in the *K-ras* oncogene according to the intake of fresh meat, meat products and fish

	**Quartile/category of intake**						
**Exposure**		**1^c^**	**2**	**3**	**4**	***P* for** **trend**	**RR (95% CI) for one s.d.** **increase in intake^d^**
*Total fresh meat*							
*Cases*							
*G>A*^+^*/G>T*^+^, *G>C*^+^							
	*Colon*	*16/16*	*16*/*12*	*31*/*13*	*19*/*15*		
	*Rectum*	*11/5*	*13*/*12*	*7*/*6*	*3*/*8*		
RR_*G*>*A*^+^_ (95% CI)	Colon	1	0.98 (0.48–1.98)	1.81 (0.97–3.40)	1.11 (0.55–2.22)	*0.34*	1.02 (0.85–1.22)
	Rectum	1	1.11 (0.49–2.51)	0.62 (0.24–1.64)	0.28 (0.08–1.03)	*0.02*	0.71 (0.55–0.93)
RR_*G*>*T*^+^, *G*>*C*^+^_ (95% CI)	Colon	1	0.75 (0.35–1.61)	0.83 (0.40–1.73)	1.00 (0.48–2.08)	*0.97*	1.01 (0.78–1.32)
	Rectum	1	2.44 (0.86–6.92)	1.01 (0.31–3.27)	1.19 (0.38–3.68)	*0.62*	0.94 (0.65–1.36)

*Beef*							
*Cases*							
*G>A*^+^*/G>T*^+^*, G>C*^+^							
	*Colon*	*16/14*	*19*/*7*	*19*/*18*	*28*/*17*		
	*Rectum*	*7/9*	*14*/*8*	*7*/*6*	*5*/*9*		
RR_*G*>*A*^+^_ (95% CI)	Colon	1	1.13 (0.57–2.26)	1.13 (0.57–2.21)	1.41 (0.74–2.66)	*0.3*	1.11 (0.91–1.36)
	Rectum	1	2.00 (0.79–5.06)	1.08 (0.37–3.11)	0.68 (0.21–2.26)	*0.25*	0.67 (0.47–0.95)
RR_*G*>*T*^+^, *G*>*C*^+^_ (95% CI)	Colon	1	0.48 (0.19–1.19)	1.26 (0.62–2.58)	1.02 (0.50–2.08)	*0.47*	0.99 (0.78–1.26)
	Rectum	1	0.81 (0.31–2.17)	0.63 (0.21–1.87)	0.82 (0.30–2.21)	*0.64*	1.06 (0.69–1.61)

*Pork*							
*Cases*							
*G>A*^+^*/G>T*^+^*, G>C*^+^							
	*Colon*	*19/13*	*23*/*18*	*25*/*12*	*15*/*13*		
	*Rectum*	*6/6*	*10*/*11*	*10*/*7*	*7*/*8*		
RR_*G*>*A*^+^_ (95% CI)	Colon	1	1.23 (0.65–2.30)	1.28 (0.69–2.40)	0.77 (0.38–1.56)	*0.53*	0.86 (0.70–1.06)
	Rectum	1	1.67 (0.60–4.66)	1.71 (0.60–4.90)	1.23 (0.40–3.77)	*0.72*	0.92 (0.68–1.25)
RR_*G*>*T*^+^, *G*>*C*^+^_ (95% CI)	Colon	1	1.43 (0.68–2.98)	0.98 (0.43–2.22)	1.07 (0.48–2.41)	*0.88*	1.10 (0.83–1.46)
	Rectum	1	1.72 (0.63–4.71)	0.99 (0.33–2.97)	1.07 (0.37–3.13)	*0.74*	1.06 (0.74–1.51)

*Minced meat*							
*Cases*							
*G>A*^+^*/G>T*^+^*, G>C*^+^							
	*Colon*	*20/12*	*15*/*18*	*19*/*16*	*28*/*10*		
	*Rectum*	*7/7*	*11*/*8*	*9*/*11*	*6*/*6*		
RR_*G*>*A*^+^_ (95% CI)	Colon	1	0.71 (0.36–1.40)	0.94 (0.49–1.81)	1.34 (0.73–2.46)	*0.24*	1.09 (0.91–1.31)
	Rectum	1	1.56 (0.61–4.01)	1.27 (0.47–3.47)	0.90 (0.30–2.72)	*0.75*	0.86 (0.61–1.22)
RR_*G*>*T*^+^, *G*>*C*^+^_ (95% CI)	Colon	1	1.44 (0.69–3.04)	1.36 (0.62–2.95)	0.83 (0.36–1.95)	*0.64*	0.89 (0.67–1.18)
	Rectum	1	1.06 (0.39–2.89)	1.51 (0.59–3.88)	0.77 (0.25–2.38)	*0.85*	0.88 (0.61–1.27)

*Liver*							
*Cases*							
*G>A*^+^*/G>T*^+^*, G>C*^+^							
	*Colon*	*56/36*	*26*/*20*				
	*Rectum*	*23/18*	*10*/*14*				
RR_*G*>*A*^+^_ (95% CI)	Colon	1	0.91 (0.55–1.49)			*0.7*	1.01 (0.82–1.26)
	Rectum	1	0.79 (0.38–1.65)			*0.53*	0.53 (0.31–0.91)
RR_*G*>*T*^+^, *G*>*C*^+^_ (95% CI)	Colon	1	1.12 (0.64–1.94)			*0.7*	1.10 (0.90–1.36)
	Rectum	1	1.45 (0.69–3.05)			*0.32*	1.18 (0.92–1.50)

*Chicken*							
*Cases*							
*G>A*^+^*/G>T*^+^*, G>C*^+^							
	*Colon*	*24/17*	*14*/*10*	*21*/*13*	*23*/*16*		
	*Rectum*	*7/6*	*11*/*10*	*8*/*9*	*7*/*7*		
RR_*G*>*A*^+^_ (95% CI)	Colon	1	0.57 (0.29–1.13)	0.84 (0.46–1.53)	0.78 (0.43–1.42)	*0.66*	0.94 (0.74–1.18)
	Rectum	1	1.59 (0.61–4.19)	1.08 (0.38–3.07)	0.85 (0.29–2.46)	*0.52*	0.88 (0.59–1.32)
RR_*G*>*T*^+^, *G*>*C*^+^_ (95% CI)	Colon	1	0.59 (0.27–1.29)	0.74 (0.36-.53)	0.81 (0.41–1.61)	*0.7*	0.95 (0.69–1.30)
	Rectum	1	1.52 (0.55–4.19)	1.36 (0.47–3.91)	0.86 (0.29–2.59)	*0.65*	0.80 (0.54–1.18)

*Other meat*							
*Cases*							
*G>A*^+^*/G>T*^+^*, G>C*^+^							
	*Colon*	*55/42*	*27*/*14*				
	*Rectum*	*20/22*	*13*/*10*				
RR_*G*>*A*^+^_ (95% CI)	Colon	1	1.33 (0.82–2.17)			*0.25*	1.12 (0.97–1.31)
	Rectum	1	1.57 (0.79–3.13)			*0.2*	1.15 (0.95–1.39)
RR_*G*>*T*^+^, *G*>*C*^+^_ (95% CI)	Colon	1	0.94 (0.50–1.77)			*0.85*	0.99 (0.76–1.29)
	Rectum	1	1.10 (0.50–2.41)			*0.81*	0.90 (0.63–1.27)

*Meat products* e							
*Cases*							
*G>A*^+^*/G>T*^+^*, G>C*^+^							
	*Colon*	*20/16*	*16*/*16*	*24*/*16*	*22*/*8*		
	*Rectum*	*6/7*	*5*/*5*	*11*/*10*	*11*/*10*		
RR_*G*>*A*^+^_ (95% CI)	Colon	1	0.79 (0.40–1.55)	1.11 (0.60–2.08)	1.08 (0.54–2.16)	*0.61*	0.98 (0.78–1.23)^e^
	Rectum	1	0.89 (0.27–2.99)	1.98 (0.69–5.62)	2.37 (0.75–7.51)	*0.07*	1.14 (0.90–1.45)^e^
RR_*G*>*T*^+^, *G*>*C*^+^_ (95% CI)	Colon	1	1.06 (0.52–2.17)	1.03 (0.50–2.11)	0.53 (0.22–1.28)	*0.2*	0.82 (0.55–1.23)^e^
	Rectum	1	0.65 (0.20–2.16)	1.16 (0.41–3.24)	1.08 (0.38–3.05)	*0.63*	1.02 (0.77–1.35)^e^

*Fish*							
*Cases*							
*G>A*^+^*/G>T*^+^*, G>C*^+^							
	*Colon*	*20/16*	*19*/*14*	*18*/*16*	*25*/*10*		
	*Rectum*	*10/9*	*5*/*5*	*16*/*10*	*2*/*8*		
RR_*G*>*A*^+^_ (95% CI)	Colon	1	1.27 (0.65–2.48)	0.81 (0.42–1.57)	1.73 (0.92–3.24)	*0.27*	1.12 (0.91–1.36)
	Rectum	1	0.61 (0.21–1.81)	1.42 (0.65–3.12)	0.28 (0.06–1.26)	*0.48*	0.78 (0.58–1.06)
RR_*G*>*T*^+^, *G*>*C*^+^_ (95% CI)	Colon	1	1.19 (0.57–2.50)	0.98 (0.48–1.98)	0.95 (0.43–2.10)	*0.8*	0.83 (0.65–1.07)
	Rectum	1	0.78 (0.25–2.40)	1.01 (0.40–2.51)	1.20 (0.45–3.22)	*0.68*	0.96 (0.72–1.28)

a Multivariate adjusted rate ratios (RRs) for age, sex, Quetelet Index (QI), smoking, energy intake and family history of CRC and their 95% confidence intervals.

b The presence of G>A transitions (*G>A^+^*) or G>T or G>C transversions (*G>T^+^, G>C^+^)* in the exon 1 fragment of the *K-ras* gene.

c Reference category/quartile of intake.

d See for one s.d. of increase based on the intake of the subcohort (Table 1).

e Rate ratios per increment of 15 g day^−1^ (s.d. in subcohort), equivalent to one standard sandwich filling.
